# Potential of Indonesian Marine Endophytic Fungi as Extracellular Enzymes Producers

**DOI:** 10.3390/jof12050374

**Published:** 2026-05-18

**Authors:** Mirah Afiza Nurazizah, Safrina Dyah Hardiningtyas, Muhammad Arief Budiman, Nurul Huda Abd Kadir, Kustiariyah Tarman

**Affiliations:** 1 Department of Aquatic Product Technology, Faculty of Fisheries and Marine Sciences, IPB University, Jl. Agatis Kampus IPB Dramaga, Bogor 16680, West Java, Indonesia; mirahafizanurazizah@apps.ipb.ac.id (M.A.N.); safrina_dyah@apps.ipb.ac.id (S.D.H.); ariefmuhammadbudiman@apps.ipb.ac.id (M.A.B.); 2 Faculty of Science and Marine Environment, Universiti Malaysia Terengganu, Kuala Nerus 21030, Terengganu, Malaysia; nurulhuda@umt.edu.my; 3 Center for Coastal and Marine Resources Studies, International Research Institute for Maritime, Ocean and Fisheries (LRI i-MAR), IPB University, Bogor 16127, West Java, Indonesia

**Keywords:** biocatalyst, mangrove, seagrass, seaweed, symbiont

## Abstract

Marine endophytic fungi inhabit the internal tissues of seaweed, seagrass, and mangroves without causing harm. These fungi are known to produce extracellular enzymes, including proteases and cellulases, which play crucial roles in various biological processes and have potential applications in diverse industrial sectors. This study aimed to screen the enzymatic potential of marine endophytic fungi, identify selected isolates, and characterize their enzyme activities. A total of 20 fungal isolates were obtained, comprising 16 isolates from seaweed, three from seagrass, and one from mangrove leaves, collected from the coastal areas of the Seribu Islands (Jakarta), Sukabumi (West Java), Nusa Dua (Bali), and the Buton Islands (Southeast Sulawesi). Screening results showed that 50% of the isolates exhibited proteolytic activity on skim milk agar, while 40% demonstrated cellulolytic activity on carboxymethylcellulose (CMC) agar. Two isolates with the highest clear zone indices for protease and cellulase activity were identified as *Penicillium citrinum* and *Fomitopsis* sp., with distinct morphological characteristics including velvety colonies and filamentous hyphal structures. The specific activities of the protease and cellulase were 5475.42 ± 2724.25 U/mg protein and 620.77 ± 607.71 U/mg protein, respectively, indicating high catalytic potential.

## 1. Introduction

Microorganisms play vital roles in ecosystems through organic matter degradation and bioactive compound production. Among them, endophytic fungi inhabiting terrestrial and aquatic environments are of particular interest due to their ecological significance and biotechnological potential. They are widely distributed in nature [1] and contribute substantially to microbial diversity when associated with algae, sponges, corals, and mangroves [2]. Marine endophytic fungi colonize host tissues without causing harm and are able to tolerate salinity, nutrient fluctuations, and temperature stress. They also enhance host resistance by inducing secondary metabolite production [3]. Their resilience under extreme conditions reflects ecological adaptation and makes them valuable resources for exploration [4,5]. Marine fungi are further recognized as prolific sources of bioactive compounds, enzymes, and other metabolites with diverse applications [6].

Marine endophytes are also prolific producers of extracellular enzymes that facilitate nutrient acquisition, defence responses, and degradation of complex compounds [7]. Microbial enzymes are advantageous due to their ease of production, scalability, and cost efficiency [8]. Enzymes reported from marine fungi include xylanase, pectinase, cellulase, chitosanase, protease, lipase, and laccase [9]. Several studies have confirmed their ability to produce amylase [10], alginate lyase, carrageenase, and agarase [11], cellulase [12,13], xylanase [14], and chitosanase [15]. Genera such as *Aspergillus*, *Penicillium*, *Fusarium*, *Nigrospora*, and *Trichoderma* are frequently isolated from marine environments and recognized as efficient enzyme producers [16,17,18]. These genera are also commonly reported as dominant marine-derived fungi, including those originating from terrestrial lineages adapted to marine habitats [6].

Enzymes derived from marine fungi possess desirable properties such as salinity tolerance, stability under extreme pH, and functionality across wide temperature ranges, reflecting their adaptation to marine habitats [19]. Examples include *Trichoderma gamsii* producing chitinase active at 5 °C [20], *Penicillium* sp. and *Alternaria* sp. producing cellulase, amylase, and DNase at low temperatures [21], and *Digitatispora marina* secreting protease stable over broad pH conditions and high salinity [22]. Other enzymes, such as cellulase and hemicellulase, degrade carrageenan [23], while protease, lipase, and xylanase hydrolyze proteins, lipids, and polysaccharides, respectively [24]. These characteristics support diverse industrial applications in pharmaceuticals, food processing, textiles, detergents, leather, and pulp and paper, with protease and cellulase being among the most widely applied [25]. Moreover, fungal enzymes contribute significantly to biorefinery and bioethanol production [26].

Despite their potential, the enzymatic diversity of marine endophytic fungi remains underexplored compared with terrestrial fungi. Many species have yet to be characterized, particularly for proteases and cellulases of industrial relevance [27]. Tropical regions such as Indonesia serve as untapped reservoirs of fungal diversity with strong prospects for discovering novel enzymes [28,29,30]. Endophytes associated with algae, seagrasses, driftwood, and mangroves are particularly promising but remain underutilized in biotechnology [31,32].

Therefore, this study aimed to screen and identify marine endophytic fungi capable of producing proteolytic and cellulolytic enzymes and to evaluate their enzymatic performance in relation to host origin and geographical variation. We hypothesized that host-associated ecological niches and geographical conditions may be associated with differences in extracellular enzyme activity among marine endophytic fungi.

## 2. Materials and Methods

### 2.1. Source of Microorganisms

This study utilized 20 fungal isolates from the collection of the Microbiology Laboratory, Department of Aquatic Product Technology, IPB University. Sampling was carried out from January to July 2022, beginning in the Seribu Islands, Northern Jakarta (Latitude: −5.735000°, Longitude: 106.601778°), followed by the coastal areas of Sukabumi, West Java Province (Latitude: −7.398111°, Longitude: 106.518583°), Nusa Dua, Bali (Latitude: −8.7954187°, Longitude: 115.2329702°), and the Buton Islands, Southeast Sulawesi Province (Latitude: −5.6031682°, Longitude: 122.8624587°).

Host plants collected at each site included *Ulva* spp., seagrass, *Sargassum* spp., *Halimeda* spp., *Gracilaria* spp., *Padina* spp., *Gelidium* spp., and mangrove leaves of *Rhizophora* sp. All samples were collected during low tide at depths of less than 1 m, with seawater salinity ranging from approximately 33–37 ppt. Healthy tissues without visible disease symptoms were selected using purposive sampling.

The initial isolation and purification of marine endophytic fungi were conducted in a previous study using standard surface sterilization and direct plating techniques [33,34]. From these isolation procedures, a total of 20 marine endophytic fungal isolates were successfully obtained and used in this study, coded as KTR3, KTR15, KTR26, KTR41, KTR42, KTR43, KTR44, KTR45, KTR46, KTR47, KTR48, KTR49, KTR50, KTR51, KTR52, KTR53, KTR54, KTR55, KTR56, and KTR57.

### 2.2. Subculturing the Marine Endophytic Fungi

The subculturing of marine endophytic fungi was performed using a potato dextrose agar (PDA) medium. PDA powder was dissolved in distilled water at a ratio of 39 g/L and then sterilized using an autoclave. The sterile PDA medium was poured into Petri dishes and allowed to cool until solidified. The fungi isolates were inoculated onto the PDA medium and then incubated at 28 °C for 7 days. The transfer of pure isolates to fresh media was conducted under aseptic conditions [35].

### 2.3. Proteolytic Activity Assay

The proteolytic activity of endophytic fungi was evaluated using skim milk agar (SMA) medium, following a modified method of [36]. A mixture of 5% skim milk and 3.9% PDA was dissolved in 300 mL of distilled water and heated to a boil, with the pH adjusted to 7. The medium was then sterilized in an autoclave, poured into Petri dishes, and allowed to solidify. The fungal isolates were inoculated onto the SMA medium and incubated at 28 °C for 7 days. The formation of a clear zone around the fungal colonies indicated proteolytic activity. The proteolytic index was calculated based on the ratio of the clear zone diameter to the colony diameter (dz/dc) [37]. Measurements were taken using a centimeter-scale ruler (China) with an accuracy of ±0.1 cm. Each measurement was repeated three times, and the average was used for analysis. The isolate exhibiting the highest enzyme activity was selected.

### 2.4. Cellulolytic Activity Assay

The cellulolytic activity was evaluated using a carboxymethyl cellulose (CMC) medium, following a modification of the method of Sari et al. [38]. The solution was prepared by mixing 1% CMC, 0.02% MgSO_4_·7H_2_O, 0.075% KNO_3_, 0.05% K_2_HPO_4_, 0.002% FeSO_4_, 0.004% CaCl_2_·2H_2_O, 0.2% yeast extract, 0.1% glucose, and 2% bacto agar in 300 mL of distilled water. The mixture was heated to a boil, adjusted to pH 7, and sterilized using an autoclave. The medium was then spread into Petri dishes and allowed to solidify. The fungi isolates were inoculated onto the CMC agar medium and incubated at 28 °C for 5 days. After incubation, 0.1% Congo red dye was applied to the surface and left for 15 min, followed by rinsing with 1% NaCl solution for another 15 min. The plates were further incubated for 48 h to enhance the formation of clear zones. The cellulolytic activity was confirmed by the presence of a clear zone surrounding the fungi colony. The cellulolytic index was determined based on the ratio of clear zone diameter to colony diameter (dz/dc) [37]. Measurements were taken to the nearest 0.1 cm using a centimeter-scale ruler. Each measurement was repeated three times, and the average was used for analysis. The isolate exhibiting the highest enzyme activity was selected.

### 2.5. Morphological Characterization of Selected Marine Endophytic Fungi

Both macroscopic and microscopic characteristics were observed to identify the marine endophytic fungi. Macroscopic observations included the surface and reverse color of the colony, colony texture (e.g., cottony, granular, powdery, or slimy), the presence of exudate droplets, and the formation of radial lines or concentric rings. The slide culture method was used for microscopic analysis to examine hyphae, spores, pigmentation, and other distinguishing features under a microscope at up to 400× magnification. These observed characteristics were then compared with relevant references for fungi identification [39,40].

### 2.6. Molecular Identification Methods

The marine endophytic fungi isolate with the highest enzymatic index for each enzyme type was identified via DNA barcoding. The PCR amplification process utilized ITS1 (5′–TCCGTAGGTGAACCTGCGG–3′) and ITS4 (5′–TCCTCCGCTTATTGATATGC–3′) primers. DNA sequencing was performed using the bidirectional sequencing method. The obtained DNA sequences were searched for similarity through the BLASTn function on the NCBI website (National Center for Biotechnology Information http://www.ncbi.nlm.nih.gov/BLAST, accessed on 22 May 2025) and analyzed for genetic relationships through phylogenetic tree analysis using MEGA 11.0 software [41]. Phylogenetic analysis was conducted using the neighbor-joining method, followed by bootstrap testing with 1000 replications [42,43].

### 2.7. Production of Crude Protease Enzyme Extract from Selected Marine Endophytic Fungi

The production of crude protease enzyme extract was carried out using selected endophytic fungi isolates exhibiting the highest proteolytic index. The production medium was prepared based on Bhagobaty et al. [44] with modifications. The medium composition consisted of 3% skim milk, 0.2% peptone, 0.1% KH_2_PO_4_, 0.05% MgSO_4_, 0.05% KCl, and 0.001% FeSO_4_. These components were dissolved in 300 mL of distilled water and heated to a boil, adjusting the pH to 7. The medium was then sterilized using an autoclave at 121 °C for 15 min. After sterilization, the selected endophytic fungi isolates were inoculated into the production medium and incubated on a rotary shaker at 170 rpm for 10 days. Following incubation, the culture was filtered to separate the fungi mycelia from the liquid phase. Then, the filtrate was centrifuged at 1700× *g*, 4 °C, for 15 min. The resulting supernatant contained the crude protease enzyme extract. The crude extract’s enzymatic activity was then subsequently assessed.

### 2.8. Production of Crude Cellulase Enzyme Extract from Selected Marine Endophytic Fungi

Production of crude cellulase enzyme extract used selected endophytic fungi isolates with the highest cellulolytic index. The process began by inoculating the isolates into a liquid CMC test medium. The medium was prepared based on Sari et al. [38] with modifications. The composition included 1% CMC, 0.02% MgSO_4_·7H_2_O, 0.075% KNO_3_, 0.05% K_2_HPO_4_, 0.002% FeSO_4_, 0.004% CaCl_2_·2H_2_O, 0.2% yeast extract, and 0.1% glucose in 300 mL of distilled water. The mixture was heated to a boil, adjusted to pH 7, and sterilized using an autoclave at 121 °C for 15 min. After sterilization, the selected endophytic fungi isolates were inoculated into the production medium and incubated on a rotary shaker at 170 rpm for 10 days. The culture was then filtered to separate the fungi mycelia from the liquid phase. The filtrate was subsequently centrifuged at 1700× *g*, 4 °C, for 15 min. The resulting supernatant contained the crude cellulase enzyme extract. The crude extract’s enzymatic activity was then assessed.

### 2.9. Protease Enzyme Activity Assay

A total of 1.5 mL of 1% casein substrate was dissolved in Tris-HCl buffer (pH 8) and pre-incubated at 37 °C for 4 min. Subsequently, 0.4 mL of crude enzyme extract was added, homogenized using a vortex mixer (VELP Scientifica Srl, Usmate Velate, Italy), and incubated at 37 °C for 20 min. The reaction was then determined by adding 2.5 mL of 0.1 M trichloroacetic acid (TCA) (10%). The mixture was further incubated at 20 °C for 10 min, and centrifuged at 2100× *g* for 10 min to further separate the supernatant from the precipitate. The absorbance of the supernatant soluble fraction was measured using a spectrophotometer (Scientific Industries, Bohemia, NY, USA) at 280 nm. Each measurement was repeated three times, and the average value was used for analysis. Protease activity was assessed following the method of Jurado et al. [42]. One unit of protease activity was defined as an increase of 0.001 absorbance units at 280 nm per minute under the assay condition. The enzymatic activity was calculated as follows: U/mL = (A280 − A0)/(0.001 × t × VE), where A280 is the absorbance at 280 nm, A0 is the absorbance of the blank solution, t is the incubation time (min), and VE is the volume of enzyme used in the reaction (mL) [45].

### 2.10. Cellulase Enzyme Activity Assay

The cellulase activity assay followed the Lorenz Miller method [46], which utilizes 3,5-dinitrosalicylic acid (DNS) reagent to quantify reducing sugars released from cellulose hydrolysis. The assay began by preparing the reaction mixture, where 0.5 mL of crude enzyme extract was mixed with 0.5 mL of 1% CMC as the substrate in a suitable buffer (pH 4.8) to maintain optimal enzymatic conditions. Then, the mixture was incubated for 30 min at 50 °C. To stop the reaction, 3 mL of DNS reagent was added, which reacted with the reducing sugars to produce a color change. The reaction mixture was then heated in a water bath (boiling, at 100 °C) for 5 min, enhancing the reddish-brown color intensity, before being rapidly cooled to room temperature. The absorbance of the solution was measured at 540 nm, using a spectrophotometer, and the amount of reducing sugars released was determined using a glucose standard curve. Each measurement was repeated three times, and the average value was used for analysis. One unit of cellulase activity (U/mL) is defined as the amount of enzyme required to release 1 µmol of glucose per minute under assay conditions. The cellulase activity was calculated as follows: U/mL = (S × DF)/(t × VE), where S is the amount of reducing sugar produced (µmol) determined from the glucose standard curve, DF is the dilution factor, t is the incubation time (min), and VE is the volume of enzyme used in the reaction (mL).

### 2.11. Protein Concentration Analysis

The protein concentration analysis was conducted based on Bradford [47]. The process began by adding 5 mL of Bradford reagent into a test tube containing 0.1 mL of the sample solution. The mixture was then incubated for 5 min to measure the absorbance using a spectrophotometer at a wavelength of 595 nm. The Bradford reagent was prepared by dissolving 10 mg of Coomassie Brilliant Blue in 5 mL of 95% ethanol and then adding 10 mL of 85% (*v*/*v*) phosphoric acid. The solution was then diluted with distilled water to a final volume of 250 mL and filtered using Whatman No. 1 filter paper before use. To prepare the standard solution, bovine serum albumin (BSA) was dissolved at a concentration of 2 mg/mL as a stock solution. Then, this stock solution was diluted with concentrations ranging from 0.1 to 1.0 mg/mL to obtain standard solutions. The standard solutions’ absorbance was measured using a spectrophotometer at 595 nm. Protein concentrations of the samples were determined based on the BSA standard curve and expressed as mg/mL of protein (BSA equivalents).

### 2.12. Data Analysis

The morphological characteristics of the marine endophytic fungi isolates were analyzed descriptively. Enzymatic screening, including proteolytic and cellulolytic activities, was evaluated semi-quantitatively based on the measurement of clear zones. Enzyme activity was further analyzed quantitatively to determine the enzyme units produced. In addition, the enzymatic index data were statistically analyzed using one-way ANOVA, followed by Tukey’s HSD post hoc test to evaluate significant differences among isolates (α = 0.05). Statistical analyses were performed using Microsoft Excel and additional statistical add-ins.

## 3. Results

### 3.1. Marine Endophytic Fungi

Endophytic fungi colonize or reside within the internal tissues of their host organisms. These fungi can originate from aquatic environments, such as seaweed, seagrass, and mangroves. We evaluated marine endophytic fungi isolates obtained from various aquatic plants. Among the 20 isolates, 16 were derived from seaweed, 3 from seagrass, and 1 from mangroves (Table 1).

### 3.2. Screening of Proteolytic Marine Endophytic Fungi

**Figure 1:**
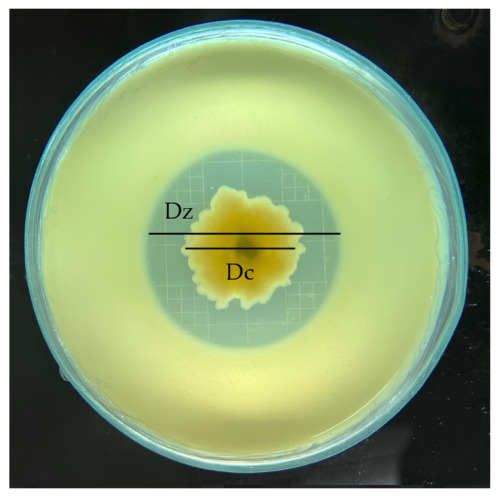
Examples of isolates that exhibited proteolytic activity **(**KTR48).

Twenty marine endophytic fungal isolates were screened for proteolytic activity. Ten isolates (50%) tested positive for protease production, as indicated by the formation of clear zones on skim milk agar (Figure 1). The highest proteolytic index was recorded for isolate KTR47 (1.60), followed by KTR48 (1.47) and KTR49 (1.42) (Table 1). The proteolytic index values ranged from 1.05 to 1.60. Isolates KTR47, KTR48, and KTR49 originated from seagrass or seaweed in Buton. Other relatively high activities were observed in KTR3 (1.41) from Sukabumi and KTR52 (1.35) from Bali. The mangrove-derived isolate KTR57 exhibited moderate activity (1.24), whereas isolates from the Seribu Islands showed no detectable protease activity. A higher enzymatic index indicates greater enzyme production by the fungal colony.

Isolate KTR47 exhibited the highest proteolytic activity but did not differ significantly from KTR48, KTR49, and KTR3, indicating comparable performance among the top-performing isolates.

### 3.3. Screening of Cellulolytic Marine Endophytic Fungi

**Figure 2:**
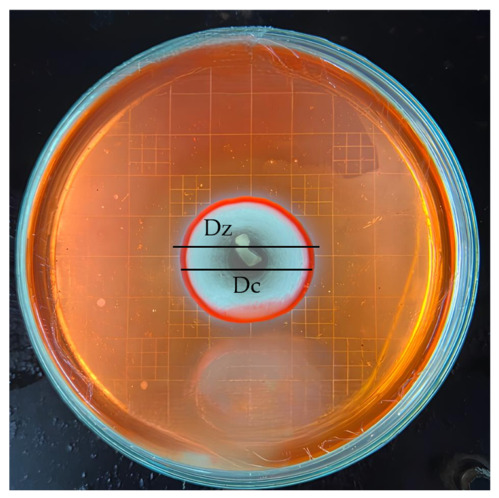
Examples of isolates that exhibited cellulolytic activity **(**KTR51).

The screening of cellulolytic activity in marine endophytic fungi revealed that 8 of the 20 isolates (40%) tested positive for cellulase production, as indicated by the formation of clear zones on carboxymethyl cellulose (CMC) agar medium (Figure 2). The highest cellulolytic indices were recorded in isolates KTR51 (1.79), KTR48 (1.62), and KTR47 (1.32) (Table 1). The cellulolytic index values in this study ranged from 1.05 to 1.79. Isolate KTR51, derived from the brown alga *Sargassum*, exhibited the highest cellulolytic activity. Seagrass-associated isolates (KTR48 and KTR47) also showed high activity, whereas the mangrove-derived isolate KTR57 showed no detectable cellulolytic activity. Geographically, isolates from Bali (KTR51) and Buton (KTR48 and KTR47) demonstrated higher cellulolytic indices compared to isolates from the Seribu Islands and Sukabumi Waters, which exhibited low to moderate or no activity.

Isolate KTR51 exhibited the highest cellulolytic activity and was significantly different from all other isolates. Isolate KTR48 showed the second-highest activity and was also significantly different from the remaining isolates. In contrast, isolates KTR47 and KTR50 did not differ significantly from each other but showed significantly lower activity than KTR51 and KTR48. These isolates also showed no significant difference compared to KTR3, indicating partial overlap among isolates with moderate activity.

### 3.4. Morphological Characteristics of Selected Marine Endophytic Fungi with Enzymatic Activity

**Figure 3:**
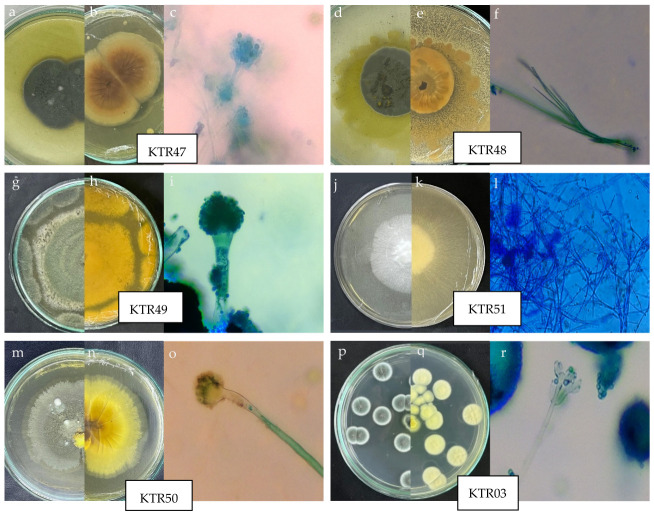
Colony morphology and microscopic characteristics of marine endophytic fungi isolates. KTR47 (**a**–**c**), KTR48 (**d**–**f**), KTR49 (**g**–**i**), KTR51 (**j**–**l**), KTR50 (**m**–**o**), and KTR03 (**p**–**r**); where the images represent colony front view, reverse view, and microscopic morphology, respectively. Colors shown in the macroscopic images represent the natural pigmentation of each isolate, while the blue coloration in microscopic images resulted from methylene blue staining.

Nine selected marine endophytic fungal isolates exhibited high enzymatic activity indices. Isolates KTR47, KTR48, and KTR49 showed the highest proteolytic indices. Isolate KTR51 exhibited the highest cellulolytic index, and isolates KTR50 and KTR3 showed higher cellulolytic indices than KTR47 and KTR48. Macroscopic and microscopic observations revealed distinct characteristics in each fungal isolate (Figure 3; Table 2 and Table 3). Based on these observations, the selected isolates were identified as belonging to the genera *Penicillium*, *Aspergillus*, *Trichoderma*, and *Mycelia*. This identification was based on differences in colony morphology, hyphal structure, and spore formation.

**Figure 4:**
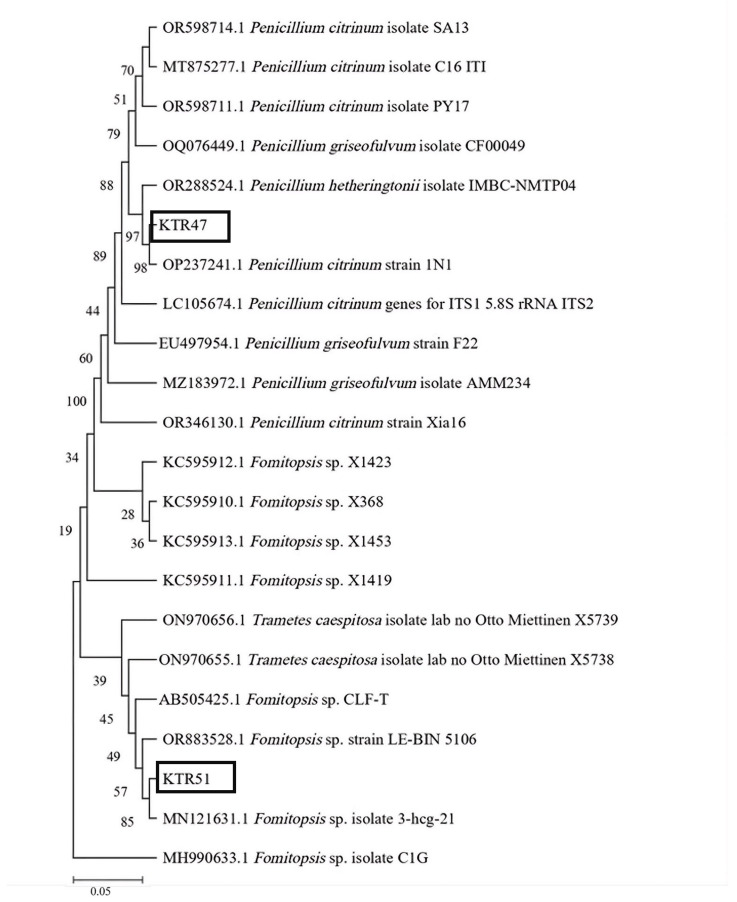
The phylogenetic tree was reconstructed using the Neighbor-Joining method (bootstrap value = 1000). Black boxes indicate the selected marine endophytic fungal isolates analyzed in this study (KTR47 and KTR51).

The isolate KTR47, which exhibited the highest proteolytic index, showed conidiophores with metulae and terminal phialides producing conidia, which are characteristic of the genus Penicillium. This isolate was further identified as *Penicillium citrinum* based on molecular analysis (Table 4, Figure 4). In addition, isolate KTR51, which showed the highest cellulolytic index, was morphologically classified as *Mycelia sterilia* due to the absence of observable spore-forming structures during microscopic examination (Table 3). However, molecular analysis identified this isolate as Fomitopsis sp. (Table 4, Figure 4). Such discrepancies are common in non-sporulating fungi, where morphological identification is limited, and molecular methods provide more reliable taxonomic resolution.

### 3.5. Molecular Identification of Selected Marine Endophytic Fungi

The two marine endophytic fungi with the highest enzymatic indices (isolate KTR47 for proteolytic activity and KTR51 for cellulolytic activity) underwent molecular identification. The ITS sequences obtained from both isolates have been deposited in GenBank under the accession numbers PX022363.1 (*Penicillium citrinum* KTR47) and PX022362.1 (*Fomitopsis* sp. KTR51). BLAST analysis showed that both isolates exhibited high sequence similarity (>99%) to reference sequences in the GenBank database (Table 4), supporting their taxonomic placement. Phylogenetic reconstruction using the Neighbor-Joining method supported the BLAST-based identifications (Figure 4). Isolate KTR47 clustered with *Penicillium citrinum* and identification was supported by a bootstrap value of 97%. Isolate KTR51 grouped with members of the genus *Fomitopsis* and its identification was supported by a bootstrap value of 57%.

### 3.6. Enzyme Activity

The crude enzyme extract activity assay was conducted to determine the enzyme’s ability to catalyze a specific chemical reaction, measured by the amount of substrate converted into product per unit time. The assay was performed using the fungi isolates with the highest proteolytic and cellulolytic indices, namely *P. citrinum* KTR47 and *Fomitopsis* sp. KTR51, respectively. Table 5 shows that the crude protease extract of *P. citrinum* KTR47 exhibited a specific activity of 5475.42 ± 2724.25 U/mg protein. On the other hand, the crude cellulase extract of *Fomitopsis* sp. KTR51 exhibited a specific activity of 620.77 ± 607.71 U/mg protein for the crude cellulase extract.

## 4. Discussion

The present study revealed distinct patterns in enzymatic activity among marine endophytic fungi, particularly in relation to host origin and isolate-specific performance. Notably, isolates associated with seagrass and macroalgae, especially those from Buton waters (KTR47, KTR48 and KTR49), consistently exhibited higher proteolytic indices compared to isolates from other locations such as isolate KTR3 from Sukabumi, KTR52 and KTR57 from Bali. This pattern may suggest a substrate-driven ecological adaptation, where fungi inhabiting plant tissues rich in structural proteins and polysaccharides develop enhanced extracellular enzyme systems to facilitate nutrient acquisition. Seagrass tissues, for instance, contain relatively higher nitrogenous compounds than many macroalgae, potentially selecting for protease-producing endophytes capable of efficiently degrading protein substrates.

Although geographic variation was observed, environmental parameters such as salinity, nutrients, and hydrodynamic conditions, known to regulate fungal metabolism and enzyme expression, were not directly measured in this study. Previous studies have shown that marine fungi modulate extracellular enzyme activity in response to salinity and environmental stress, which can affect enzyme production and catalytic efficiency [48,49]. Therefore, although the observed geographic trends are consistent with ecological expectations, further environmental characterization is needed to establish a causal relationship.

The proteolytic index values observed in this study (1.09–1.60) fall within the typical range reported for marine endophytic fungi, confirming that only a small proportion of isolates express detectable protease activity. Similar findings have been reported in previous studies, where proteolytic activity was limited to a small proportion of isolates and varied significantly depending on host species and origin [50,51]. This variability is likely governed by genetic regulation and substrate availability, as extracellular enzyme production is often inducible and dependent on environmental cues.

The specific protease activity of the crude enzyme isolate *Penicillium citrinum* KTR47 was 5475.42 ± 2724.25 U/mg, which substantially exceeds the values reported in comparable studies [52,53]. This difference can be interpreted from both methodological and biological perspectives. 

From a methodological perspective, the definition of enzyme activity used in this study based on the increase in absorbance at 280 nm during casein hydrolysis may result in higher calculated values compared to assays based on tyrosine release or other standard protease quantification methods. Variations in assay conditions, substrate concentrations, and quantification approaches are known to affect reported enzyme activities, making direct comparisons between studies extremely difficult [54]. From a biological perspective, marine fungi are exposed to fluctuating and often extreme environmental conditions, including high salinity, osmotic pressure, and nutrient limitation. These stresses have been reported to be associated with the activation of biosynthetic gene clusters and may enhance the production of extracellular enzymes with improved catalytic properties [55,56].

The relatively high standard deviation observed in protease and cellulase specific activity may be attributed to inherent variability in shake-flask cultivation systems. Although all isolates were cultured under identical nominal conditions, small differences in microenvironmental factors such as oxygen transfer, nutrient distribution, and fungal growth dynamics can occur between individual Erlenmeyer flasks. These variations are known to influence microbial growth rates and enzyme production, particularly for extracellular enzymes such as proteases, which are highly sensitive to culture conditions [51,57]. Previous studies have reported that enzyme production in filamentous fungi is strongly dependent on growth phase and environmental conditions, and even minor variations during fermentation can lead to significant differences in enzyme yield [57,58]. Additionally, shake-flask systems are inherently heterogeneous, and variability between replicates has been widely documented in protease production studies [59,60]. The use of crude enzyme extracts may also contribute to variability due to the presence of mixed proteins and potential interfering compounds affecting absorbance measurements. Therefore, the observed standard deviation likely reflects biological and process-related variability rather than experimental error.

The strong cellulolytic activity observed in the *Sargassum*-derived isolate (KTR51) may be related to the high structural polysaccharide content of the brown alga, which may select for cellulase-producing endophytes. Similarly, the increased activity in the seagrass-associated isolate supports previous findings that cellulose-rich plant tissues stimulate cellulase production [12]. The absence of detectable activity in isolates from mangroves suggests that cellulase production among marine endophytic fungi is host-dependent. Geographical differences in cellulolytic activity may be associated with variations in environmental factors such as salinity, nutrient availability, and hydrodynamic conditions, which are known to influence the expression of fungal metabolites [61].

Quantitative enzyme assays further highlight the biotechnological potential of selected isolate *P. citrinum* KTR47 produced a protease with an activity of 2184.00 ± 1010.22 U/mL and a specific activity of 5475.42 ± 2724.25 U/mg protein, values significantly higher than those reported by [52], who obtained only 22.44 ± 0.71 U/mL from *P. citrinum* isolated from animal housing soil. This difference may be influenced by the content of compounds in the isolates’ original substrates, such as total nitrogen or amino acid content. This would provide different biosynthetic pathways in marine fungi, directly stimulating secondary metabolism and protease enzyme production [8]. These results offer potential for broad applications in the pharmaceutical, animal feed, and food industries, highlighting the potential of *P. citrinum* KTR47 as a marine protease producer with possible industrial relevance [27].

In contrast, the cellulase activity of *Fomitopsis* sp. KTR51 (620.77 ± 607.71 U/mg) was moderate compared to terrestrial references, as higher activity has been reported in *F. meliae* CFA2 [62] and *Fomitopsis* sp. RCK2010 [63], although a much lower activity was observed in *Penicillium oxalicum* R4 (6.35 U/mL) [13], indicating substantial interspecific variability. The moderate activity of the present isolates may reflect differences in substrate preferences, as marine *Fomitopsis* strains may be more adapted to degrading algal polysaccharides than lignocellulose. Nevertheless, marine cellulases are often reported to exhibit salt tolerance and stability under extreme conditions; however, these properties were not evaluated in the present study and require further investigation. Despite this limitation, such enzymes continue to attract attention for their potential applications in industrial bioprocessing [64,65] and their broad applications in biofuel production, wastewater treatment, textiles, animal feed, and the food industry [25,66,67].

The coexistence of proteolytic and cellulolytic activities in several isolates further illustrates the metabolic performance of marine endophytic fungi. This multifunctionality is consistent with previous reports showing that marine fungi can produce diverse enzyme systems in response to substrate availability and environmental conditions [18,68]. This adaptability enhances their ecological role in nutrient cycling and underscores their potential as sources of industrial enzymes.

A limitation of this study is that the stability and performance of the enzymes under varying salinity, pH, and temperature conditions were not evaluated. Therefore, the present work should be considered an initial screening and preliminary activity assessment. Further studies are required to characterize enzyme stability and tolerance under different conditions to confirm their adaptation to marine environments and potential industrial applications.

Overall, the results of this study indicate that marine endophytic fungi, particularly those associated with seagrass ecosystems, are a promising source of extracellular enzymes with high catalytic activity. The potential ability of these organisms to function under saline and dynamic environmental conditions, as reported for marine-derived fungi, may suggest adaptation to marine environments; however, this remains to be experimentally verified.

## 5. Conclusions

This study demonstrated that marine endophytic fungi isolated from diverse coastal habitats produce extracellular protease and cellulase enzymes, with *Penicillium citrinum* KTR47 showing the highest proteolytic activity and *Fomitopsis* sp. KTR51 exhibiting notable cellulolytic potential. These findings highlight the functional diversity of marine endophytes and their relevance as alternative enzyme sources. Despite these promising results, the study is limited to preliminary screening and activity assessment under single conditions. Therefore, further research is required to optimize enzyme production and to characterize their biochemical properties, including stability and performance under varying environmental conditions. Such studies will be essential to evaluate their applicability in industrial processes.

## Figures and Tables

**Table 1:** Sources of marine endophytic fungi isolates and the enzymatic activity.

Isolate Code	Host Plants	Sample Location	Enzymatic Index ^1,2^	
			Proteolytic	Cellulolytic
KTR41	*Ulva* sp.	Seribu Islands	-	-
KTR42	*Ulva* sp.	Seribu Islands	-	1.05 ± 0.01 ^e^
KTR3	*Vaucheria* sp.	Sukabumi Waters	1.41 ± 0.23 ^ab^	1.19 ± 0.04 ^cd^
KTR43	*Halimeda* sp.	Sukabumi Waters		-
KTR15	*Halimeda* sp.	Sukabumi Waters	1.09 ± 0.02 ^b^	-
KTR44	*Ulva* sp.	Sukabumi Waters	1.34 ± 0.02 ^ab^	-
KTR26	*Ulva* sp.	Sukabumi Waters	-	-
KTR45	*Padina* sp.	Buton, Sulawesi Tenggara	-	1.15 ± 0.06 ^d^
KTR46	*Cladophora* sp.	Buton, Sulawesi Tenggara	-	
KTR47	Seagrass (unknown species)	Buton, Sulawesi Tenggara	1.60 ± 0.08 ^a^	1.32 ± 0.09 ^c^
KTR48	Seagrass (unknown species)	Buton, Sulawesi Tenggara	1.47 ± 0.39 ^ab^	1.62 ± 0.01 ^b^
KTR49	*Sargassum* sp.	Buton, Sulawesi Tenggara	1.42 + 0.03 ^ab^	
KTR50	*Caladophora* sp.	Buton, Sulawesi Tenggara	1.17 ± 0.08 ^b^	1.31 ± 0.02 ^c^
KTR51	*Sargassum* sp.	Nusa Dua, Bali	1.19 ± 0.05 ^b^	1.79 ± 0.08 ^a^
KTR52	*Solieria* sp.	Nusa Dua, Bali	1.35 ± 0.05 ^ab^	1.17 ± 0.05 ^d^
KTR53	Seagrass (unknown species)	Nusa Dua, Bali	-	-
KTR54	*Gellidella* sp.	Nusa Dua, Bali	-	-
KTR55	*Sargassum* sp.	Nusa Dua, Bali	-	-
KTR56	*Gracilaria* sp.	Nusa Dua, Bali	-	-
KTR57	*Rhizophora mucronata* leaf	Nusa Dua, Bali	1.24 ± 0.09 ^b^	-

^1^ Enzymatic index: ratio of clear zone diameter to colony diameter (dz/dc). ^2^ Different superscript letters indicate statistically significant differences among isolates (Tukey’s HSD test, α = 0.05).

**Table 2:** Colony characteristic of marine endophytic fungi.

Code	Surface Colony	Reverse Colony	Structure Elevation	Elevation	Pattern	Exudate Drops	Radial Line	Concentric Circle
KTR47	Dark greenish brown	Yellowish brown	Velvety	Umbonate	Radiate	-	-	-
KTR48	Dark greenish brown	Yellowish brown	Velvety	Umbonate	Radiate	√	-	-
KTR49	Green with a yellowish tint	Yellow	Velvety	Umbonate	Spread	-	√	√
KTR51	White	White to cream	Cottony	Umbonate	Zonate	-	-	-
KTR50	Dark greenish black	Brownish black	Velvety	Umbonate	Zonate	-	√	√
KTR03	Dark green	Yellow to cream	Velvety	Rugose	Spread	-	√	√

Note: √ indicates presence, while - indicates absence of the characteristic.

**Table 3:** Microscopic characteristics of marine endophytic fungi.

Isolate	Spore	Shape	Hyphae	Characteristic	Taxonomic Identification
KTR47	Conidia	Cylindrical	Septate	Hyaline conidiophores, erect, branched penicillately at the apexes with verticillate metulae and terminal phialides	*Penicillium*
KTR48	Sporangia	Cylindrical	Septate	Branched hyphae without distinct structures such as phialides or conidiophores	*Mycelia sterilia*
KTR49	Conidia	Globose	Septate	Straight, upright conidiophores with globose conidia that are greenish-blue and attached to the phialides	*Aspergillus*
KTR51	Sporangia	Cylindrical	Septate	Branched mycelial structure, does not show spore formation, but appears as a denser and tangled hyphal network	*Mycelia sterilia*
KTR50	Conidia	Globose	Septate	Simple, upright conidiophores with globose conidia at the tips that have phialides	*Aspergillus*
KTR03	Conidia	Subglobose	Septate	Hyaline conidia, subglobose in shape, highly branched conidiophores with a broad conidial production area, phialides indicating conidia production	*Trichoderma*

**Table 4:** Identification of species using BLAST analysis.

Code of Isolate	Description	Evaluate	Query Cover (%)	Identity (%)	Accession Number
	* Penicillium citrinum * strain 1N1	0.0	98	99.46	OP237241.1
	* Penicillium citrinum * isolate SA13	0.0	97	99.63	OR598714.1
	* Penicillium griseofulvum * isolate CF00049	0.0	97	99.63	OQ076449.1
KTR47	* Penicillium citrinum * strain Xia16	0.0	97	99.63	OR346130.1
	* Penicillium hetheringtonii * isolate IMBC-NMTP04	0.0	97	99.63	OR288524.1
	* Penicillium citrinum *	0.0	97	99.63	LC105674.1
	* Penicillium griseofulvum * isolate AMM234	0.0	97	99.63	MZ183972.1
	* Penicillium citrinum * isolate C16 ITI	0.0	97	99.63	MT875277.1
	* Penicillium griseofulvum * strain F22	0.0	97	99.63	EU497954.1
	* Penicillium citrinum * isolate PY17	0.0	96	99.63	OR598711.1
KTR51	* Fomitopsis * sp. isolate C1G	0.0	99	98.85	MH990633.1
	* Trametes caespitosa * isolate lab no Otto Miettinen X5738	0.0	99	98.85	ON970655.1
	* Fomitopsi * s sp. X1419	0.0	99	98.85	KC595911.1
	* Fomitopsis * sp. CLF-T	0.0	99	98.85	AB505425.1
	* Fomitopsis * sp. strain LE-BIN 5106	0.0	99	99.69	OR883528.1
	* Trametes caespitosa * isolate lab no Otto Miettinen X5739	0.0	99	99.69	ON970656.1
	* Fomitopsis * sp. X1453	0.0	99	99.54	KC595913.1
	* Fomitopsis * sp. X1423	0.0	99	99.23	KC595912.1
	* Fomitopsis * sp. X368	0.0	99	99.38	KC595910.1
	* Fomitopsis * sp. isolate 3-hcg-21	0.0	100	99.23	MN121631.1

**Table 5:** Activity of crude extracellular enzyme extract from marine endophytic fungi.

Isolate	Produced Enzyme	Enzyme Activity (U/mL)	Protein Concentration (mg/mL)	Enzyme Specific Activity (U/mg Protein)
*P. citrinum* KTR47	Protease	2184.00 ± 1010.22	0.402 ± 0.020	5475.42 ± 2724.25
*Fomitopsis* sp. KTR51	Cellulase	200.00 ± 192.87	0.325 ± 0.006	620.77 ± 607.71

## Data Availability

The data presented in the study are available on request from the corresponding author.
